# Supplementary figures and images for: Maximal inhibitory effect of MOV10 on LINE-1 retrotransposition requires both the MOV10/LINE-1 association and granule formation
Source: PLoS Genet. 2025 May 23;21(5):e1011709. doi: 10.1371/journal.pgen.1011709 (PMC12140422; doi:10.1371/journal.pgen.1011709)

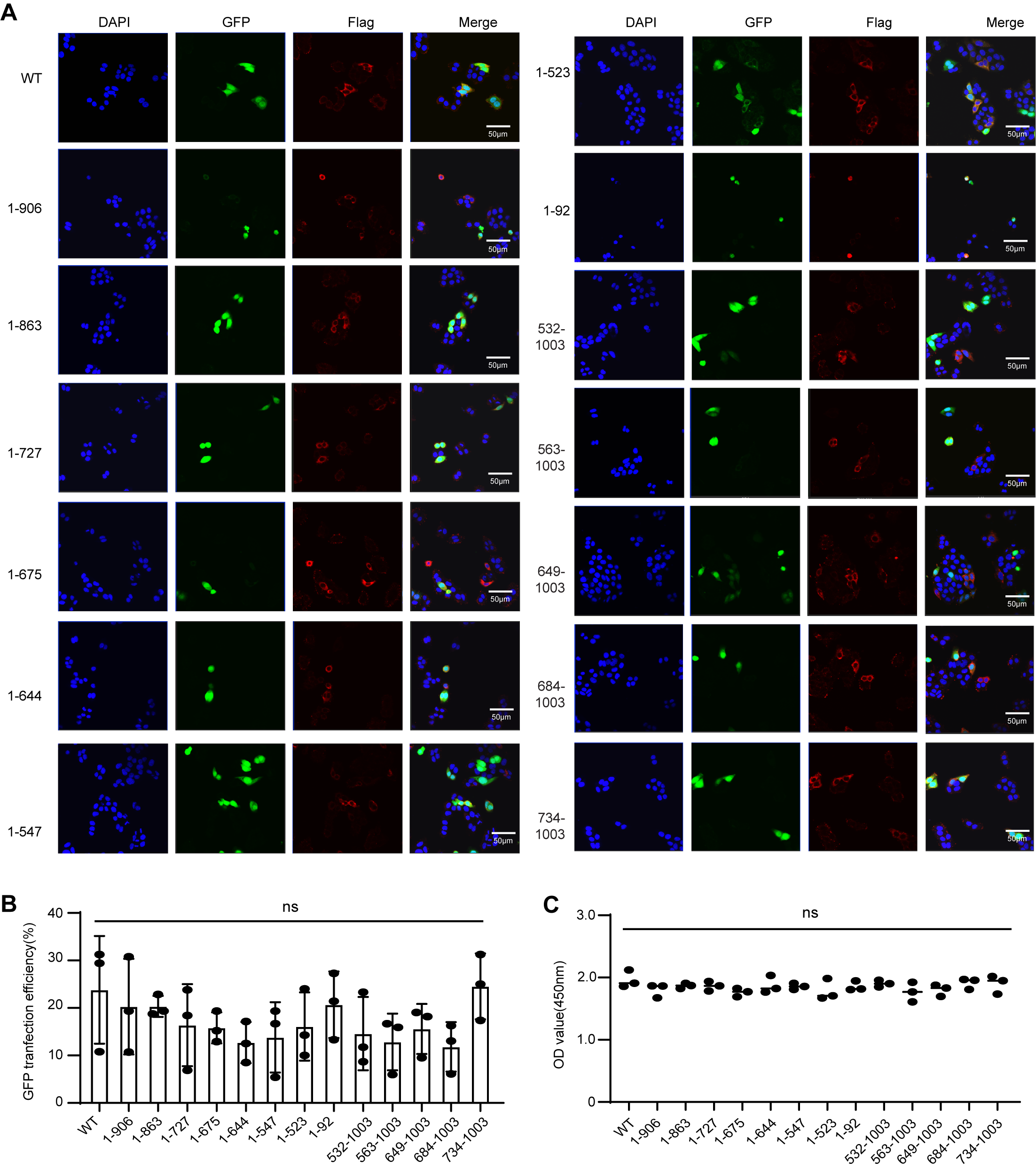

Supplement: S1 Fig — (A) HeLa cells were co-transfected with 500ng full length MOV10 or MOV10 mutants and 500ng GFP plasmid. Immunofluorescence confocal microscopy was performed to determine the subcellular localization of DAPI (blue), MOV10(Red) and GFP(Green). (B)Transfection efficiency of GFP transfected with MOV10 or MOV10 mutants. Error bars indicate SD, P-value was determined using ordinary one-way ANOVA test. (C). HeLa cells were transfected with 500ng full length MOV10 or MOV10 mutants, CCK-8 assays were performed to assess cell proliferation at 48h. The data from three independent experiments were summarized in the bar graph. Error bars indicate SD, P-value was determined using ordinary one-way ANOVA test. ns means no significance. (TIF) [file pgen.1011709.s001.tif]

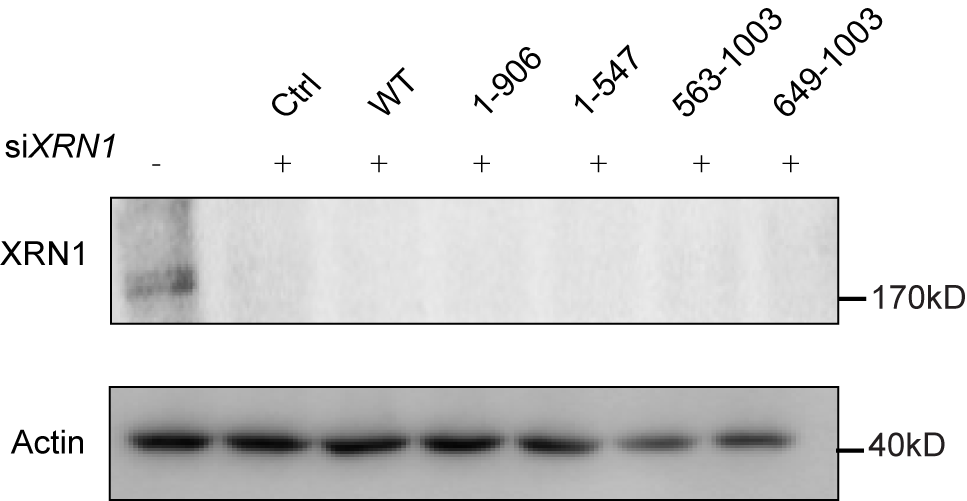

Supplement: S2 Fig — Western blots were probed with antibodies for the detection of XRN1and Actin expression. (TIF) [file pgen.1011709.s002.tif]

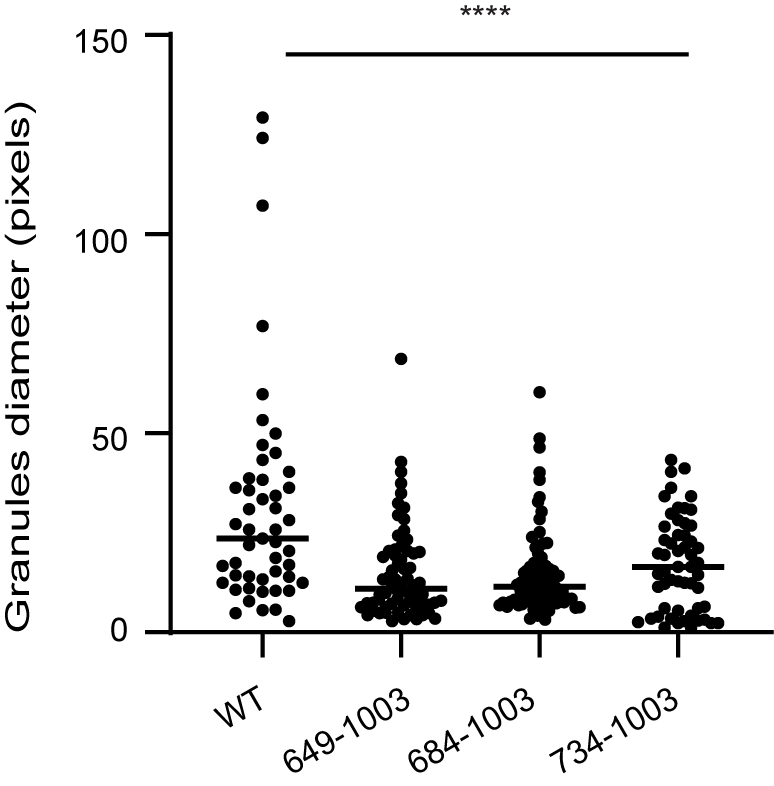

Supplement: S3 Fig — Granules size of full length MOV10 and MOV10 mutants. The area of granules formed by full-length MOV10,649-1003,684-1003 and 734-1003 (n = 49,66,77,55). Error bars indicate SD, P-value was determined using ordinary one-way ANOVA test. ****P < 0.0001. (TIF) [file pgen.1011709.s003.tif]

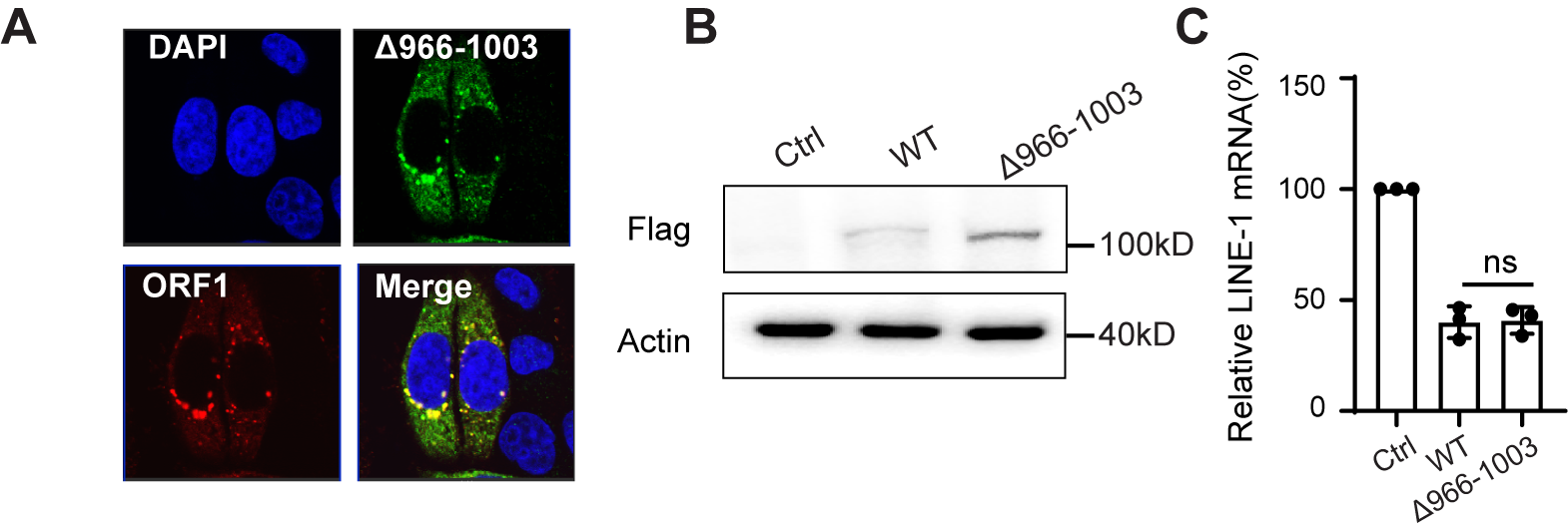

Supplement: S4 Fig — (A)Confocal images cytoplasmic localization of MOV10 Δ966-1003 and ORF1p using anti-Flag and anti-ORF1p antibodies. (B) Western blots were probed with antibodies for the detection of MOV10 WT, Δ966-1003 (anti-Flag) and Actin expression. (C)RNA isolated from HeLa cells was quantified by qPCR and normalized to GAPDH expression. The data from three independent experiments were summarized in the bar graph. Error bars indicate SD, P-value was determined using ordinary one-way ANOVA test. ns means no significance. (TIF) [file pgen.1011709.s004.tif]

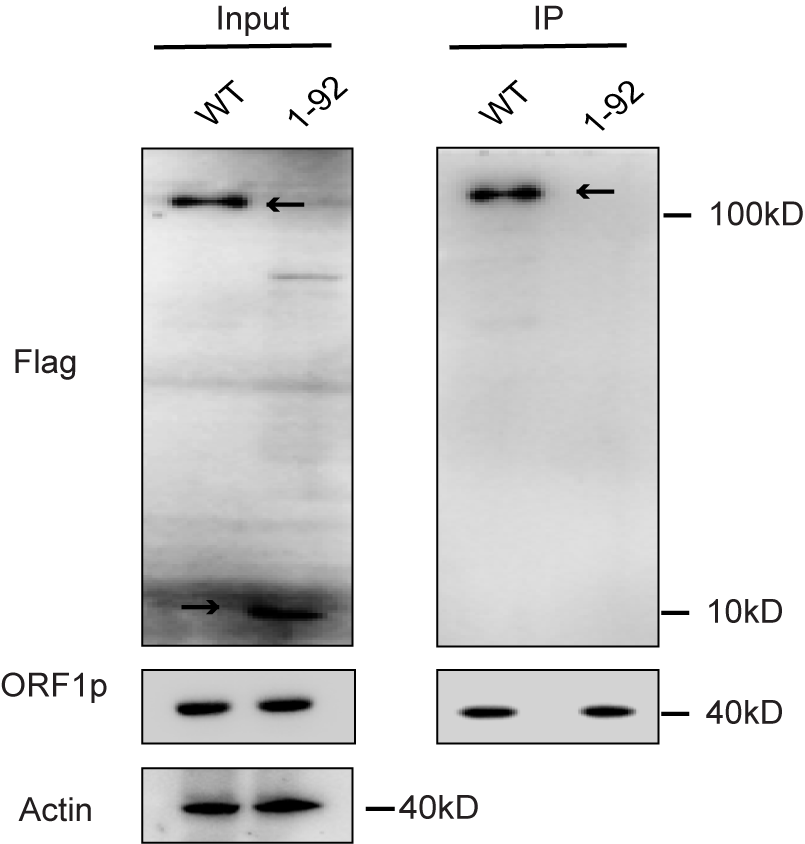

Supplement: S5 Fig — Input and anti-Flag IPs were subjected to immunoblot analysis using antibodies against Flag, ORF1p and Actin. (n = 3 biological replicates). (TIF) [file pgen.1011709.s005.tif]

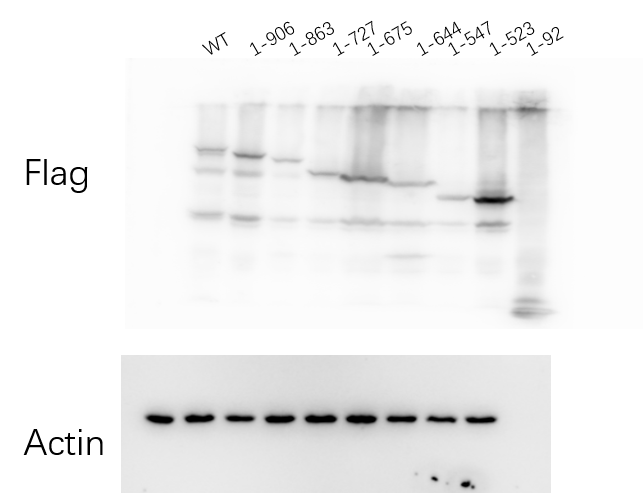

Supplement: S2 Data — Source data for western blots in all figures. (ZIP) [file pgen.1011709.s007.zip › S2 data. Source data for Western bolts/Fig 1B.tif]

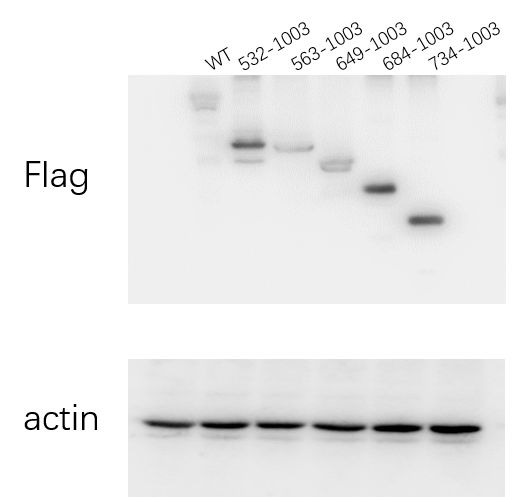

Supplement: S2 Data — Source data for western blots in all figures. (ZIP) [file pgen.1011709.s007.zip › S2 data. Source data for Western bolts/Fig 2B.tif]

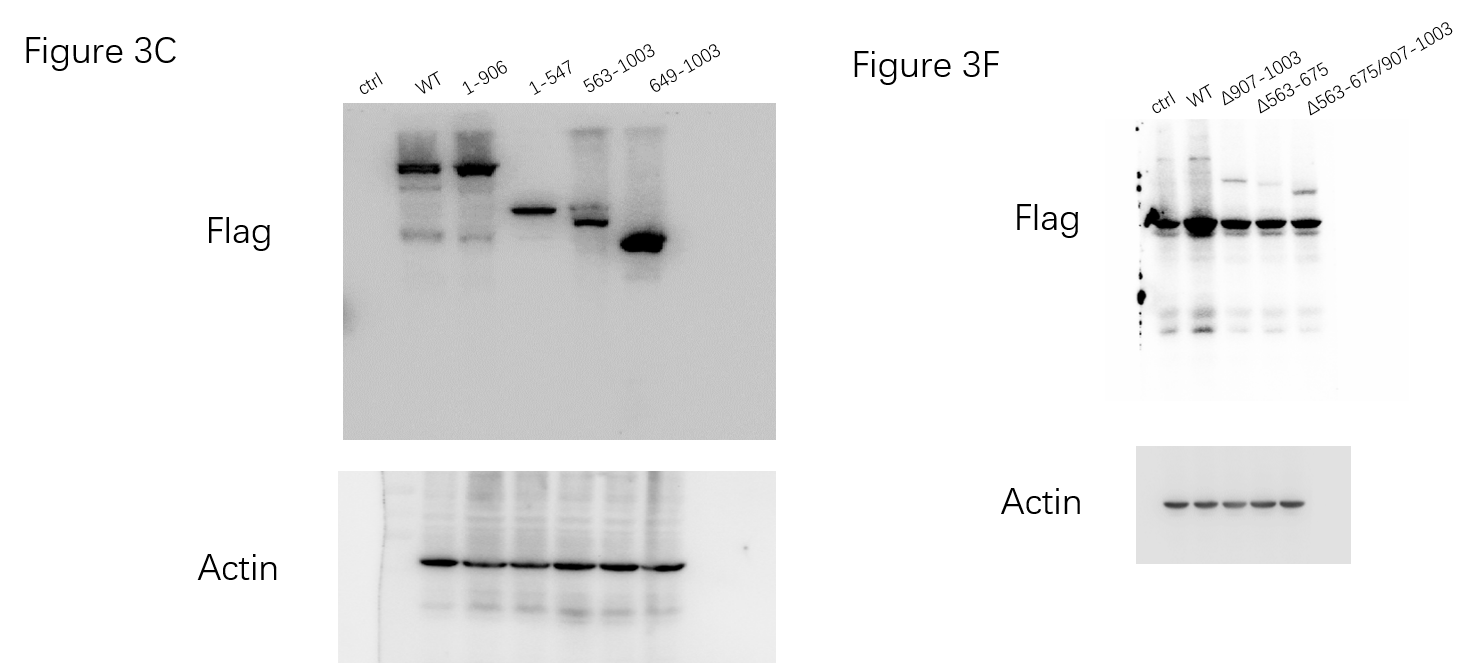

Supplement: S2 Data — Source data for western blots in all figures. (ZIP) [file pgen.1011709.s007.zip › S2 data. Source data for Western bolts/Fig 3C and 3F.tif]

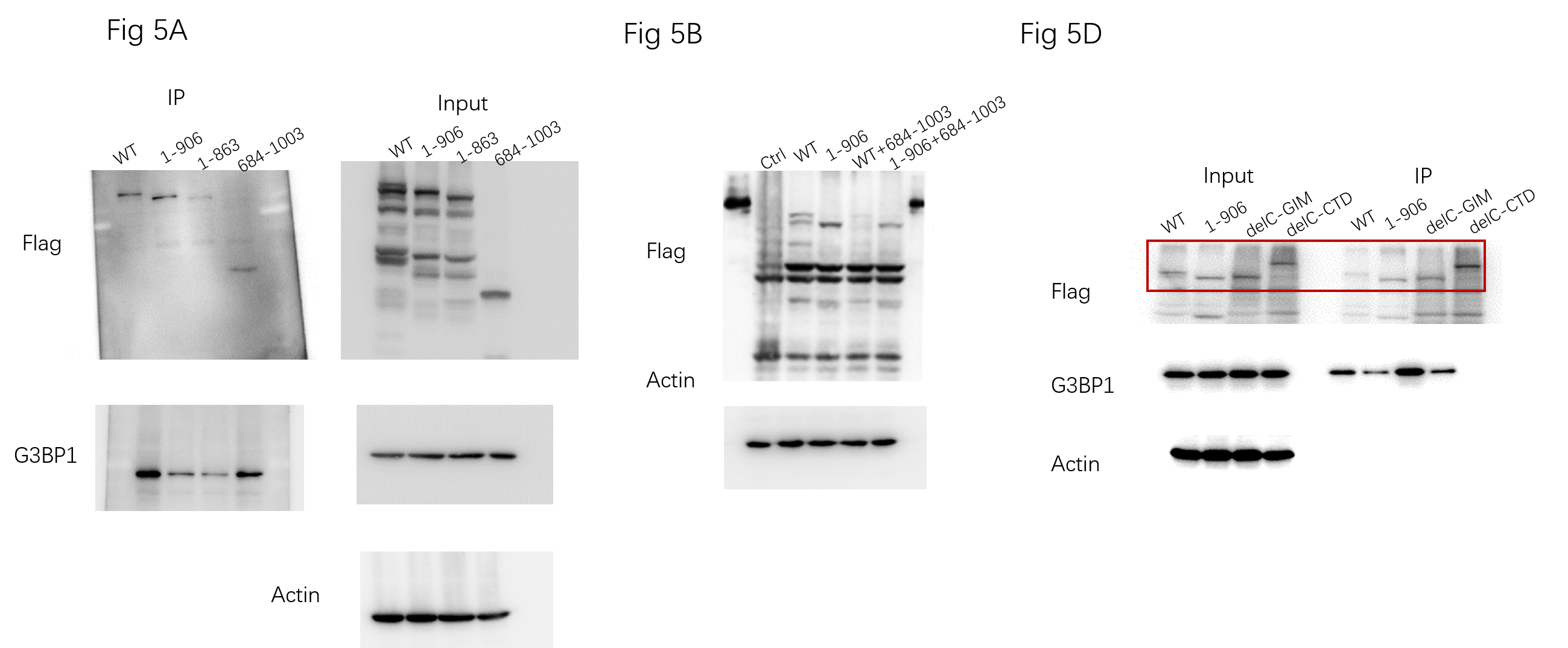

Supplement: S2 Data — Source data for western blots in all figures. (ZIP) [file pgen.1011709.s007.zip › S2 data. Source data for Western bolts/Fig 5A, 5B and 5D.tif]

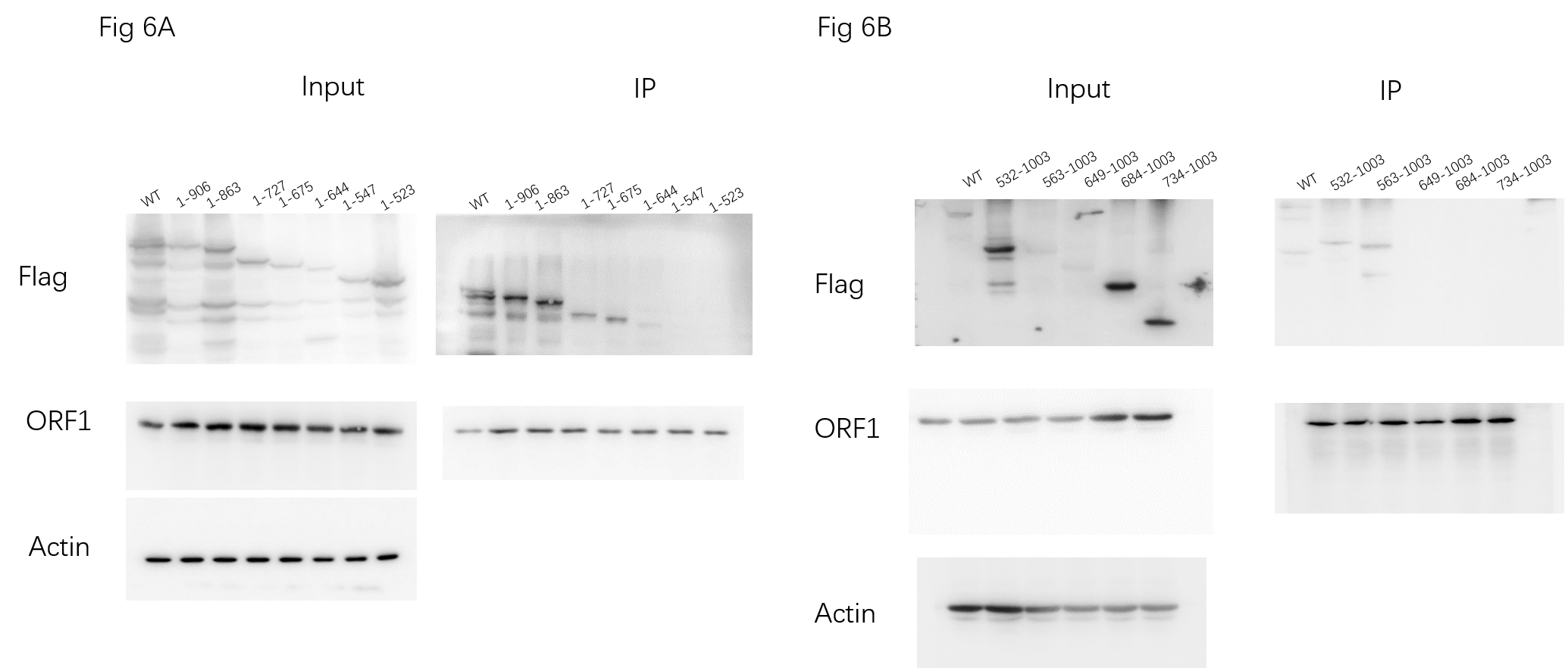

Supplement: S2 Data — Source data for western blots in all figures. (ZIP) [file pgen.1011709.s007.zip › S2 data. Source data for Western bolts/Fig 6A and 6B.tif]

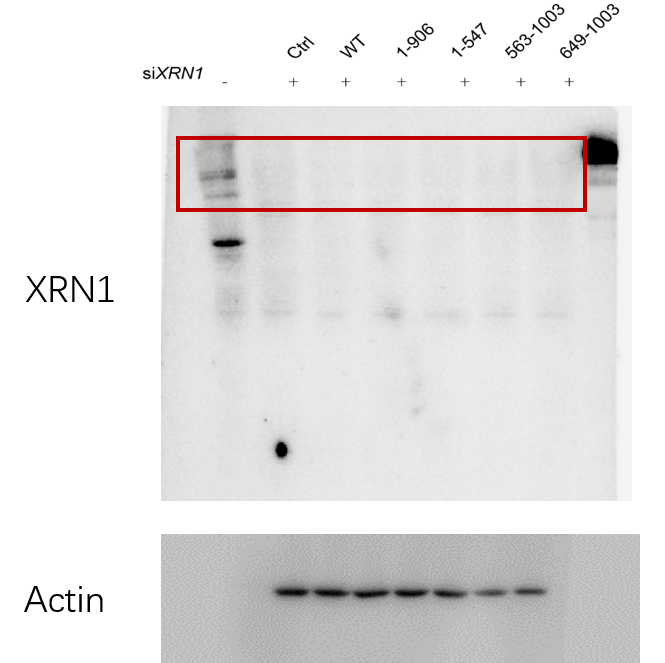

Supplement: S2 Data — Source data for western blots in all figures. (ZIP) [file pgen.1011709.s007.zip › S2 data. Source data for Western bolts/S2 Fig.tif]

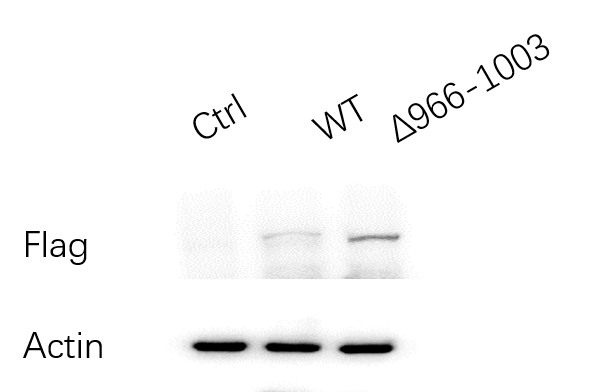

Supplement: S2 Data — Source data for western blots in all figures. (ZIP) [file pgen.1011709.s007.zip › S2 data. Source data for Western bolts/S4B Fig.tif]

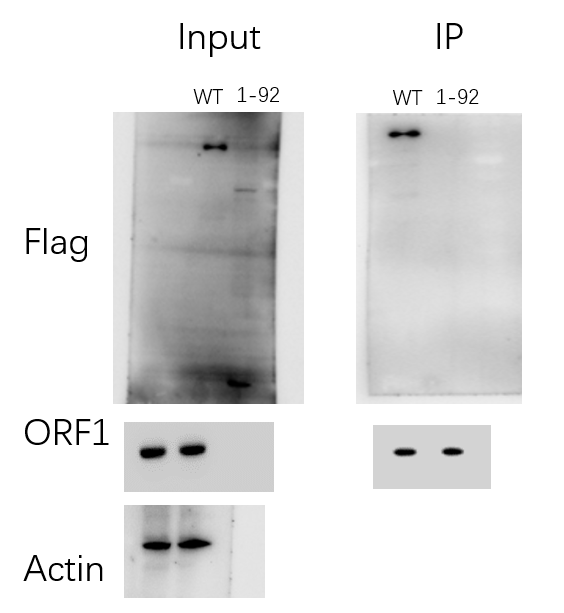

Supplement: S2 Data — Source data for western blots in all figures. (ZIP) [file pgen.1011709.s007.zip › S2 data. Source data for Western bolts/S5 Fig.tif]
