# Supplementary material for: Maximal inhibitory effect of MOV10 on LINE-1 retrotransposition requires both the MOV10/LINE-1 association and granule formation
Source: PLoS Genet. 2025 May 23;21(5):e1011709. doi: 10.1371/journal.pgen.1011709 (PMC12140422; doi:10.1371/journal.pgen.1011709)
Supplement: Attachment — Submitted filename: 20250314-Response.docx [file pgen.1011709.s009.docx]

***Reviewer #1: This is an interesting and important study that seeks to understand how the RNA helicase MOV10 suppresses LINE-1 retrotransposition. LINEs are the only active autonomous mobile DNA in the human genome, thus, their regulation is important to study. Creation of the MOV10 constructs, their use in the retrotranposition assay, the LINE-1 RNA degradation experiments and expression in cells made for a detailed and compelling study. The work identified two important regions in MOV10, “extended motif II” and the distal C-terminus (aa 907-1104) that cooperate to achieve maximal LINE 1 inhibition. Their role in puncta formation and colocalization with ORF1 was very compelling. Some of the conclusions were not correct, though, and must be addressed. There were also some mistakes and omissions that need to be fixed, as described below.***

***Major points.***

1. ***There is a problem with the title: this paper does not formally show phase-separation, rather it demonstrates puncta/granule formation. It seems the title should be changed to reflect that.***

**Response:** We are grateful for the reviewer's suggestion. We have changed the title to “Maximal inhibitory effect of MOV10 on LINE-1 retrotransposition requires both the MOV10/LINE-1 association and granule formation”. To align with the title, we also change “LLPS” in line 32 to “granules”, “LLPS” in line 34 to “large granules formation”, “LLPS” in line 38 to “granules”, “LLPS” in line 126 to “granules”, “LLPS” in line 328 to “granules”, “LLPS” in line 456 to “large granules”, “LLPS” in line 462 to “granules”.

2. ***Figure 1. the legend does not match the panels: C and E are switched***

**Response：**We apologize for the mistake and have corrected the legend of Figure 1C and 1E.

3. ***In general, all figure legends need to include the statistical tests used and the number of replicates tested.***

**Response:** We have added the statistical tests used and the number of replicates tested to all figure legends.

4. ***The statement describing Fig. 4B says that the puncta are of smaller size. The sizes need to be quantified.***

**Response:** We thank the reviewer for the suggest. We have now conducted a statistical analysis of size of granules formed by full-length MOV10 and ORF1, MOV10 mutant 649-1003, 684-1003 and 734-1003 alone, and discovered that they are larger in size compared to the granules formed when only MOV10 are present. The results were supplemented as S3 Fig in the revised manuscript.

**Response Figure 1.** Granules size of full length MOV10 and MOV10 mutants. The area of granules formed by full-length MOV10,649-1003,684-1003 and 734-1003 (n=49,66,77,55). Error bars indicate SD, P-value was determined using ordinary one-way ANOVA test. ****P<0.0001.

5. ***Please give the citation supporting the sentence on pg. 18 “It is worthy noted that a disordered sequence (amino acids 966-1003) was predicted within the C-terminal domain, which represents a key feature for promoting LLPS.”***

**Response：**The predicted disorder sequence (amino acids 966-1003aa) of MOV10 was published in Uniprot, which was predicted using Sequence Analysis Methods for automatic annotation.

We have now added the information in the sentence as the following:

*“It is worthy noted that a disordered sequence (amino acids 966-1003) was predicted within the C-terminal domain published in Uniprot, which represents a key feature for promoting LLPS.” (Page 17)*

6. ***The authors need to cite the following papers as showing evidence that MOV10 regulates LINE retrotransposition:***

***Goodier JL, Cheung LE, Kazazian Jr HH. MOV10 RNA helicase is a potent inhibitor of retrotransposition in cells. PLoS Genet. 2012;8(10):e1002941.***

***Skariah, G, Seimetz, J, Norsworthy, M, Lannom, MC, Kenny, PJ, Elrakhawy, M, Forsthoefel, C, Drnevich, J, Kalsotra, A, Ceman, S. (2017). Mov10 suppresses retroelements and regulates neuronal development and function in developing brain. BMC Biology. 15(1):54 PMID:28662698***

**Response:** We have now added the two references in the text as evidence that MOV10 regulates LINE-1 retrotransposition in the revised manuscript. (Page 4, line 88)

7. ***In Figure 5C, there is a “CIM” domain in the schematic while the text refers to the Caprin G3BP1 interacting domain as “GIM.” This is confusing. Please rectify this in either the text of figure legend.***

**Response:** We have corrected “CIM” to “GIM” in the schematic and figure legends.

8. ***A statement in the Discussion is not correct: “These results suggest that association of MOV10 with G3BP1 is required but insufficient for the granule formation, and the C-terminal domain of MOV10 shall involve other functions required for promoting LLPS.” There was no evidence in this manuscript that association with G3BP1 is required for MOV10 to be found in puncta. If there is evidence elsewhere, it should be cited here. In fact, the opposite seems to be true: when the authors engineered MOV10 with the GIM/CIM motif and forced association with G3BP1 (Fig. 5D), it did not lead to puncta formation (Figure 5E). What this work does show is that the C-terminal region from 906 to 1004 is required for MOV10 to form puncta, which is interesting and should be discussed.***

**Response:** We agree with the reviewer and have now removed the words “required” from the sentence and added more discussion on the potential role of the C-terminal domain of MOV10.

*“According to the MOV10 structure published on UniProt, the region 966-1003 is predicted to be a disordered sequence, which is one of the critical factors for the formation of phase-separated structures. Our findings demonstrated that deletion of amino acids 966-1003 in MOV10 does not compromise its functional capacity. Specifically, the truncated MOV10 variant maintains the ability to form large cytoplasmic granules and complete activity comparable to the full-length MOV10(S3 Fig), suggesting an importance role of amino acids 906-966 in puncta formation. Among the many characterized phase-separating proteins, common molecular features, such as multivalency[**47**], intrinsically disordered regions[**41**], and structured RNA-binding[**48**] have been demonstrated to play critical roles in driving condensation. The interaction between RNA-binding proteins and RNA represents another pivotal element in the assembly of phase-separated granules. Prior research has demonstrated that RNA facilitates the formation of LLPS granules[**49**], yet an overabundance of RNA can result in their disintegration[**48**]. The C-terminal region (amino acids 907-1003) of MOV10 is presumed to be instrumental in RNA binding, orchestrating the formation of phase-separated granules via its engagement with RNA and a deeper exploration is required to elucidate the exact mechanisms at play.” (Page 23)*

9. ***Also in the Discussion that is not correct: because the authors see the N-terminal 92 amino acids of MOV10 in the nucleus, they infer that it has an NLS. That is incorrect: proteins less than 45 kDa in size can diffuse into the nucleus. This statement needs to be amended or removed.***

**Response:** Agree. We have now removed the following sentences from the revised manuscript.

*“In addition, the image analysis showed that 1-92, 532-1003 and 563-1003 were predominantly located in the nucleus, whereas wild type and other mutated MOV10 were found in the cytoplasm (Figure 4). This suggests two nuclear localization signals (NLS) within the first 92 and 563-649 amino acids, and one cytoplasmic retention signal (CRS) within the amino acid 92-523, all of which awaits further investigation.”*

10. ***There are a number of grammatical errors including a paucity of articles (the, a, etc..) and incorrect verb tenses. This should be corrected.***

**Response:** We appreciate the reviewer’s comments and have done our best to refine the text accordingly.

***Reviewer #2: In the manuscript ”Maximal inhibitory effect of MOV10 on LINE-1 retrotransposition requires both the MOV10/LINE-1 association and phase separation”, the authors investigate how MOV10 inhibits L1 retrotransposition by employing the use of MOV10 deletion mutants. Their data suggests that the MOV10 extended motif II (563-675aa) and the CTD (906-1003aa) cooperate to achieve “maximal” MOV10-mediated inhibition of L1 activity. They propose that motif II is involved in mediating interactions between MOV10 and L1, and that the CTD is required for the association of MOV10 with G3BP1 and the formation of liqid-liqid phase separation (LLPS), which promotes the “maximal” inhibition of L1 activity by MOV10.***

***The use of MOV10 deletion mutants is logical strategy to approach the difficult problem of unraveling how MOV10 inhibits L1 retrotransposition. However, the findings of this study do not provide significant insight into this mechanism beyond what has already been reported. In addition, some of the main conclusions are not well supported by the data and some of the experiments lack important controls. The manuscript also lacks experimental details and is poorly referenced. Please see below for detailed comments:***

1. ***It is unclear what the authors mean by “maximal” inhibitory effect. The term “maximal” is not defined clearly and therefore confusing. The data show that several of the MOV10 CTD mutants inhibit L1 (figure 1) to a significant effect in their assays. For example, MOV10 mutant 1-863 inhibition is ~80% compared to WT MOV10, which is indeed a very strong inhibitory effect. In addition, other CTD mutants still inhibited L1 quite strongly. Notably, the CTD mutants dd not form cytoplasmic foci (LLPS?), which suggests that foci formation is not strictly required for inhibition. Together, these data do not seem consistent with the title and the author’s model that the MOV10 CTD/G3BP1 interaction and LLPS is required for MOV10-mediated inhibition.***

**Response：**Thank the reviewer for the comment. We would like to explain that the maximal activity represents the complete activity of full length MOV10.

We agree with the reviewer that foci formation is not strictly required for the inhibition of LINE-1 by MOV10. Indeed, compared with the N-terminal truncations, the C-terminal deletions impaired MOV10's anti-LINE-1 activity to a lesser degree, resulting in a 30% reduction compared to its maximum potential (A significant difference is denoted by one asterisk). This suggests that granule formation is one of the conditions required for MOV10 to achieve its maximal activity, while it is not strictly required as binding of MOV10 to LINE-1 does.

2. ***The authors did not indicate how they monitored transfection efficiency or how they monitored for potential toxic side effects of the transfected MOV10 plasmids in their retrotransposition assays. Variations in transfection efficiencies and/or toxicity of the MOV10 plasmids could result in differences in retrotransposition efficiency (for example see Fig. 1C and 1D) and/or variations in protein expression levels for the different MOV10 plasmids (e.g., Fig. 1B). The authors must monitor transfection efficiency and also monitor for potential toxic side effects of the exogenous MOV10 plasmids in their retrotransposition assays.***

**Response：**Thank the reviewer for the valuable comment**.** To assess the transfection efficiency of various MOV10 constructs, we co-transfected each plasmid with a GFP-expressing plasmid respectively into HeLa cells (Response Figure 2A). After 48 hours of transfection, the cells were analyzed using confocal microscopy. The results demonstrated comparable GFP fluorescence intensity across all experimental groups, indicating consistent transfection efficiency among different samples. Consequently, the observed variations in protein expression levels can be attributed to differential expression efficiencies or stability of the mutated proteins (Response Figure 2B).

On the other hand, we utilized the CCK-8 assay to evaluate the cytotoxicity of exogenous MOV10 plasmids. The results revealed that proliferation of HeLa cells transfected with different MOV10 plasmids were significantly reduced compared to the control group, suggesting the minimal cytotoxicity of exogenous MOV10 plasmids (Response Figure 2C).

**Response Figure 2. A.** HeLa cells were co-transfected with 500ng full length MOV10 or MOV10 mutants and 500ng GFP plasmid. Immunofluorescence confocal microscopy was performed to determine the subcellular localization of DAPI (blue), MOV10(Red) and GFP(Green). **B.** Transfection efficiency of GFP transfected with MOV10 or MOV10 mutants. **C.** HeLa cells were transfected with 500ng full length MOV10 or MOV10 mutants, CCK-8 assays were performed to assess cell proliferation at 48h. The data from three independent experiments were summarized in the bar graph. Error bars indicate SD, P-value was determined using ordinary one-way ANOVA test. ns means no significance.

We have now added the new data in the S1 Fig, and described the results as the following:

*“To minimize the influence of transfection efficiency and cell cytotoxicity from various MOV10 plasmids on the cell colony system, we employed a GFP co-transfection approach to monitor transfection efficiency (S1A Fig) and utilized the CCK8 assay (S1B Fig) to evaluate cell cytotoxicity. The results demonstrated that the transfection efficiency of GFP co-transfected with different MOV10 plasmids remained highly consistent (S1C Fig), and their cytotoxicity was negligible compared to the control group.” (Page 8)*

3. ***The manuscript is not adequately referenced; many factual statements do not have references or contain the wrong reference. Here are several of examples from the introduction although there are more instances throughout the manuscript:***

***a. Line 48: ORF1 chaperone activity should have at least the following references: (DOI: 10.1128/MCB.21.2.467-475.2001 and DOI: 10.1073/pnas.0809964106)***

***b. Line 53: “TPRT” requires reference to Luan et al. (DOI: 10.1016/0092-8674(93)90078-5)***

***c. The CMV-L1-neoRT plasmid should be referenced and attributed to the Heidmann lab (DOI: 10.1038/74184). Line 112***

***d. The author should at a minimum cite Moran et al, (DOI: 10.1016/s0092-8674(00)81998-4) for the retrotransposition assay.***

***e. Line 66: Goodier et al. should be cited for the fact that MOV10 inhibits L1 retrotransposition (DOI: 10.1371/journal.pgen.1002941)***

**Response：**Thank the reviewer for the suggestions. We have corrected the wrong reference and added relevant references in the revised manuscript as following:

a) Two references (DOI: 10.1128/MCB.21.2.467-475.2001 and DOI: 0.1073/pnas.0809964106)1 were cited in line 69,

b) The references (DOI: 10.1016/0092-8674(93)90078-5) was added in line 74,

c) The reference (DOI: 10.1038/74184) was cited in line 138,

d) The reference (DOI: 10.1016/s0092-8674(00)81998-4) was cited in line 140,

e) Two references (DOI: 10.1371/journal.pgen.1002941 and DOI: 10.1186/s12915-017-0387-1.) were added in line 88.

4. ***The authors propose that MOV10 interaction with G3BP1 and LLPS formation is involved in inhibition of L1 retrotransposition. Notably, Goodier et al. (DOI: 10.1371/journal.pgen.1002941) first showed that ORF1p and MOV10 localize to cytoplasmic granules suggesting that cytoplasmic foci or granules could be involved in MOV10 inhibition. Subsequent studies examining other host factors that inhibit L1 have reported similar phenomenon, although the connection remains unclear between inhibition and cytoplasmic foci or granules. Since the authors propose that G3BP1 is involved in these foci, and somehow connected with L1 inhibition, it would be helpful to know the proportion of ORF1p/MOV10 foci that actually contain G3BP1. Also, one could ask the question whether MOV10 inhibits L1 in cells lacking G3BP1 or whether ORF1p/MOV10 foci form in cells that lack G3BP1. Do the authors think the foci they are seeing are stress granules?***

**Response:** Thank the reviewer for the valuable comment. In fact, our previous research (PMC10481665) revealed that when MOV10 and LINE-1 are co-overexpressed in cells, they co-localize with G3BP1 and form larger granular structures compared to when they are expressed individually. The results indicate that these granules contain G3BP1 (Response Figure 3A). Although the mechanism by which G3BP1 mediates the formation of stress granules involving LINE-1 inhibitory factors remains unclear, as a scaffold protein of stress granules, we hypothesize that it plays a significant role. Supporting this, our earlier studies showed that siRNA-mediated knockdown of G3BP1 disrupts the large granular structures formed by MOV10 and LINE-1 and reduces MOV10’s anti-LINE-1 activity (Response Figures 3B and 3C).

Furthermore, we propose that the granules formed by MOV10 and LINE-1 are stress granules. Cells experience stress when LINE-1 is overexpressed or dysregulated, leading to the formation of stress granules. This is supported by the co-localization of MOV10 /LINE-1 with G3BP1, a well-established marker of stress granules. However, the composition of stress granules varies depending on the type of cellular stress, and the underlying mechanisms require further investigation.

**Response Figure 3.** **A**. G3BP1 was co-localized with DMLC-containing granule in the presence of MOV10. Confocal images showing cytoplasmic localization of MOV10, LINE-1 ORF1p and G3BP1 using anti-Flag, anti-ORF1p and anti-G3BP1 antibodies. **B.** Confocal images showing that silencing *G3BP1* by siRNA. **C.** Silencing the endogenous *G3BP1* restored the level of LINE-1 RNA in the presence of MOV10(n=3 biological replicates).

5. ***Throughout the manuscript figures, there is no indication of how many times experiments were repeated and what statistics were used to determine significance (p-values). The authors need to indicate how many times experiments were repeated and how results were calculated as well as the statistical methods used to analyze results if they are going to show p-values.***

**Response：**Thank the reviewer for the suggestion. We have added the information in the figure legends.

6. ***In figure 1 it is unclear what the bars and asterisks indicate in the graphs in 1D and 1E.***

**Response：**Thank the reviewer for the suggestion. We have added the information to the figure legends. Error bars indicate SD, P-value was determined using ordinary one-way ANOVA test. ns means no significance; ****P<0.0001.

7. ***The source of the ORF1p antibody should be listed in the methods.***

**Response:** We have listed the source of ORF1p in the methods (Page 28).

8. ***Please indicate MW size markers (kDa) on all western blot images.***

**Response:** We have added the MW size markers (kDa) on all western blot images.

***Reviewer #3: This is an interesting and thorough study of how MOV10 restricts LINE-1 (L1) retrotransposition in human cells. Testing a series of MOV10 C-terminus and N-terminus mutants in retrotransposition assays, the authors nicely show which parts of the MOV10 helicase inhibit LINE-1 mobility and interact with G3BP1, which has shown be shown to be involved in phase separation. The presence of LINE-1 ORF1p in granules, which has been studied extensively in relation to MOV10, is shown here to rely on the G3BP1 interacting C-terminus of MOV10. The data also suggest that the extended motif II of the MOV10 helicase interacts with the LINE-1 RNP (although it isn't defined for certain whether this is more closely with ORF1p or ORF2p). When combined, interactions between the extended motif II and the C-terminus of the MOV10 helicase with the LINE-1 RNP and G3BP1, respectively, are necessary for MOV10 to exert its maximal effect as an LINE-1 inhibitor. These conclusions are reasonable based on the data presented. Generally I found the presentation was clear and I could follow the text easily. Although questions remain, this work makes a valuable advance on our understanding of how MOV10 inhibits LINE-1.***

***Moderate issue:***

***Figure 5A - how reproducible is the gel showing interactions with G3BP1. Although I agree with the interpretation, the bands are relatively faint. It would be useful in supplemental to show two more replicates of this experiment. I realise that this will take additional work but it seems it important to show what parts of the MOV10 helicase are interacting with G3BP1.***

**Response:** We thank the reviewer for the valuable suggesting. We have now provided two more replicates of the experiment and replaced the original figure 5A with one new set data showing strong bands but slight background.

**Response Figure 4.** Interactions among endogenous G3BP1 and MOV10 or MOV10 truncations. MOV10 was overexpressed in HEK293T cells. Input and anti-Flag IPs were subjected to immunoblot analysis using antibodies against G3BP1, Flag and actin.

**Minor issues:**

***line 53 - please cite PMID: 1722352 alongside the Feng et al EN paper.***

***Science. 1991 Dec 20;254(5039):1808-10. doi: 10.1126/science.1722352.***

**Response：**Thank the reviewer for the comment. We have added the reference in line 70.

***line 58 - I would cite a review summarising diseases caused by LINE-1 insertions (e.g. PMID: 27158268 or a more recent one).***

**Response：**We have added the references (PMID:27158268 and PMID: 31515540) in line 80.

***line 67 - mentioning DNA methylation and other factors limiting LINE-1 transcription in human cells, I think it would be prudent to cite PMID: 31230816 and PMID: 38309261 here.***

**Response：**We have added the references (PMID: 31230816 and PMID: 38309261) in line 89 and 90.

***line 73 - it would be collegiate to cite the previous work from Goodier et al on MOV10 (PMID: 23093941)***

**Response：**We have added the reference (PMID: 23093941) in line 95.

***Results - from the outset it could be stated that the retrotransposition assays were done in HeLa cells (although I do note that is said in the figures)***

**Response:** Thank the reviewer for the comment. We have stated the retrotransposition assays were done in HeLa cells. (Page 6, line 142)

***Nomenclature - interactions with ORF1 are noted where the authors are referring to the protein ORF1p.***

**Response:** We have corrected ORF1 to ORF1p.

***Figure 5B - the histogram doesn't really line up with the labels of the blot above (assume x-axis has those labels).***

**Response:** We have adjusted the histogram to confirm the columns line up with the blot.

***line 375 - the Discussion uses the terminology "full" and "maximal" to describe LINE-1 inhibition. This is somewhat open to interpretation because the data show "maximal" inhibition is achieved, not "full" as there is still significant mobility despite the presence of untruncated MOV10.***

**Response：**We have changed “Full” to “maximal” in line 447 and 456.

***Discussion - there are some mentions of previous studies that are uncited in the text; please provide these references.***

**Response：**We have now added the references in the Discussion part in the revised manuscript.

***Methods - please provide a reference for the CMV-L1-neo^RT^ reporter.***

**Response:** We have cited the reference for the CMV-L1-neo^RT^ in the text.

Geoff Faulkner (University of Queensland)

***Reviewer #4: This study was designed based on previous research that human MOV10 recruits DCP2 to decap LINE-1 RNA through liquid-liquid phase separation (LLPS), thereby inhibiting LINE-1 retrotransposition. In this study, the authors further explored the functional domains of MOV10 for LINE-1 inhibition and underlying mechanisms of MOV10 function. They found that MOV10 binds to LINE-1 through its extended motif II (563-675aa), which is the primary mechanism by which it inhibits LINE-1 activity. Furthermore, the C-terminal domain of MOV10 (906-1003aa) enhances LLPS, thereby maximizing MOV10's inhibitory effect on LINE-1 replication. This is an interesting and valuable study.***

***Major Points:***

1. ***The study emphasizes the crucial role of MOV10's extended motif II (563-675aa) and C-terminal in LINE-1 inhibition. However, the authors did not directly truncate or construct these two regions separately or combined for further investigation, leading to a lack of more direct evidences. In addition, to avoid the conformational effect of large truncated mutants, the authors can construct plasmids of MOV10 mutant (Δ563-675aa), MOV10 mutant (Δ563-675aa and Δ906-1003 ) to verify their effects on LINE-1.***

**Response：**We appreciate the constructive comments of the reviewer. We have now constructed MOV10 mutants Δ563-675 and Δ563-675/Δ906-1003, and investigated their effect on LINE-1 retrotransposition. In brief, HeLa cells were transfected with full length MOV10, Δ563-675, Δ906-1003 or Δ563-675/Δ906-1003 with L1-CMV-neo^RT^. After 48 hours, quantitative PCR (qPCR) was performed to measure the levels of LINE-1 RNA, while Western blotting was employed to detect the corresponding protein expressions. The results showed that MOV10 Δ563-675 and Δ563-675/Δ906-1003 failed to reduce the level of LINE-1 RNA. This suggests that both of the two mutants exhibit no anti-LINE-1 activity, whereas Δ906-1003 present almost 40% inhibition activity (Response Figures 5A and 5B). Additionally, cellular distribution of the mutants was examined. The image showed that mutant Δ563-675 formed smaller granules but did not co-localize with ORF1p, and mutant Δ563-675/Δ906-1003 displayed a diffuse distribution pattern throughout the cytoplasm (Response Figure 5C).

We have now added the new data in the Figure 3 and Figure 4, and described the results as the following:

*“To avoid the conformational effect of large truncated mutants, two MOV10 mutants, namely Δ563-675 and the double mutant Δ563-675/Δ907-1003，were constructed(Fig 3E). HeLa cells were then transfected with either full-length MOV10, the single mutant Δ907-1003 (1-906), the single mutant Δ563-675, or the double mutant Δ563-675/Δ907-1003, with the L1-CMV-neoRT vector. The results revealed that both the Δ563-675 mutant and the double mutant Δ563-675/Δ906-1003 lost their anti-LINE-1 activity. In contrast, the Δ906-1003 mutant retained partial inhibitory activity against LINE-1, which is consistent with the result of MOV10 truncations (Figs 3F and 3G).”* *(Page 11)*

“*Additionally,* *Imaging analysis demonstrated that the Δ563-675aa mutant formed smaller cytoplasmic granules, which notably failed to co-localize with ORF1p, and the double mutant Δ563-675aa/Δ907-1003 exhibited a diffuse cytoplasmic distribution, lacking the granular structures (Fig 4C).” (Page 14)*

**Response Figure 5. A.** Schematic of the structure of MOV10 Δ907-1003, Δ563-675 or Δ563-675/Δ907-1003. Numbers indicate AA positions. **B.** HeLa cells were transfected with CMV-L1-neo^RT^ and full length MOV10, MOV10 Δ563-675, Δ907-1003 or Δ563-675aa/Δ907-1003. Western blots were probed with antibodies against Flag and actin expression. **C.** RNA isolated from HeLa cells was quantified by qPCR and normalized to GAPDH expression. The data from three independent experiments were summarized in the bar graph. **D.** Confocal images cytoplasmic localization of MOV10 Δ907-1003, Δ563-675 or Δ563-675/Δ907-1003 and ORF1p using anti-Flag and anti-ORF1p antibodies. Error bars indicate SD, P-value was determined using ordinary one-way ANOVA test. ns means no significance；*P< 0.05; ***P<0.001; ****P<0.0001.

2. ***In Figure 5, when exploring the function of MOV10's C-terminal domain, the authors mention the disordered region (966-1003aa) in line 310 of the main text. It might be better to truncate this disordered region for further study.***

**Response：**We have truncated this disordered region by constructing MOV10Δ966-1003. The results showed that with deleting 966-1003aa, MOV10 co-localized with ORF1p also and formed larger granules, indicating that 966-1003aa contributed minimally to the formation of large granules (Response Figure 6A). Data of qPCR revealed that Δ966-1003 maintained complete anti-LINE-1 activity as full length MOV10(Response Figures 6B and 6C), suggesting that 906-966aa act as important role in granules formation.

We have now added the new data in the S4 Fig, and described the results as the following:

“*It is worthy noted that a disordered sequence (amino acids 966-1003) was predicted within the C-terminal domain published in Uniprot, which represents a key feature for promoting LLPS. To confirm the role of 966-1003aa in granules formation, the construct MOV10Δ966-1003 was used. The results demonstrated that deletion of the 966-1003aa region did not impair MOV10's ability to co-localize with ORF1p and form larger granules (S4A Fig), indicating that this region contributes minimally to the formation of large granules. qPCR data revealed that the Δ966-1003 mutant retained full anti-LINE-1 activity (S4B and 4C Figs), comparable to that of the full-length MOV10. These findings suggest that the C terminal amino acids 906-966 region plays a critical role in the formation of granules.” (Page 17)*

**Response Figure 6. A.** HeLa cells were transfected with CMV-L1-neo^RT^ and full length MOV10 or MOV10Δ966-1003. Western blots were probed with antibodies for the detection of MOV10 WT, Δ966-1003 (anti-Flag) and actin expression. **B.** RNA isolated from HeLa cells was quantified by qPCR and normalized to GAPDH expression. The data from three independent experiments were summarized in the bar graph. **C.** Confocal images cytoplasmic localization of MOV10 1-966 and ORF1p using anti-Flag and anti-ORF1p antibodies.

3. ***In Figure 6A, the immunoprecipitation (IP) results show that pulled down MOV10 wild-type (lane 1) is significantly less than the truncated proteins (lanes 2-6), suggesting that the interaction between MOV10 WT with ORF1 is weaker than that of truncated mutants, which is odd. Moreover, they didn’t examine truncated 1-92, so it is just to infer its interaction with ORF1. Additionally, in Figure 6B, the input results show considerable differences in protein expression levels among the truncated mutants (lanes 3-4 are notably weaker). Better to quantify the bands by ImageJ. Similar issues appear in other figures such as Figure 2B, which might affect the results.***

**Response：** We sincerely thank the reviewer for the valuable comments. Firstly, the enhancement of the interaction between 563-1003 and ORF1 may be due to either the increased exposure of ORF1 binding site (amino acids 563-675) in MOV10 after the deletion of amino acids 532-563, or higher expression level than that of wild type in cytoplasm, while the mechanism awaits further investigation. To address the confusion regarding Figures 6A, we have replaced these figures with data from repeated experiments and performed quantitative analysis using ImageJ to ensure the reliability of our results (Response Figure 7A and 7B).

Secondly, we have conducted additional experiments to investigate the potential interaction between truncation 1-92 and ORF1p. The results demonstrate that no interaction exists between these two protein (Response Figure 7C).

We have now added the new data in the S5 Fig, and described the results as the following:

“*We also investigated the potential interaction between the nuclear-localized 1-92 and ORF1p, and no interaction was observed as expected (S5 Fig).*”*(Page 20)*

**Response Figure 7.** **A.** Interactions among MOV10 C-terminal mutants and ORF1p in HEK293T cells. **B.** Interactions among MOV10 C-terminal mutants and ORF1p in HEK293T cells. **C.** Interactions among MOV10 1-92 and ORF1p in HEK293T cells. Input and anti-Flag IPs were subjected to immunoblot analysis using antibodies against Flag, ORF1p and actin. (n=3 biological replicates)

As the suggestion of the reviewer, we quantify the bands of related western blot by ImageJ.

***Minor Points:***

1. ***In Figure 1D (line 143) and Figure 1E (line 144), the descriptions are reversed.***

**Response:** We apologize for the mistake and have corrected the legend of Figure 1C and 1E.

2. ***In Figure 3D, the first lane for XRN1 is present, but the first lanes for FLAG and Actin are missing. It would be better to maintain consistency.***

**Response:** Thank for the reviewer’s comments. The XRN1 band serves as evidence to confirm that XRN1 has been successfully knocked down in the experimental cells, ensuring the prevention of decapped RNA degradation. To maintain consistency, we separated the XRN1 band from the panel and added the control protein actin to create a new panel as S2 Fig.

Response Figure 8. Endogenous XRN1 was knocked down by siRNA. Western blots were probed with antibodies for the detection of XRN1 and actin expression.

3. ***In Figure 5B, the non-specific bands are too strong. Arrows should be used to indicate the positions of the MOV10 truncations in the figure.***

**Response:** Thank the reviewer for the advice. We have used arrows to indicate the target bands.

4. ***The intensity of MOV10 (WT) and its truncations binding to G3BP1 in Figures 5A and 5D can be quantified using ImageJ.***

**Response：**Thank the reviewer for the comment. The bands of G3BP1 in Figures 5A and 5D were quantified using ImageJ, and the corresponding quantitative values (IP/Input) were indicated beneath panel.
