# Supplementary material for: Maximal inhibitory effect of MOV10 on LINE-1 retrotransposition requires both the MOV10/LINE-1 association and granule formation
Source: PLoS Genet. 2025 May 23;21(5):e1011709. doi: 10.1371/journal.pgen.1011709 (PMC12140422; doi:10.1371/journal.pgen.1011709)
Supplement: Attachment — Submitted filename: 20250420-response.docx [file pgen.1011709.s010.docx]

***Reviewer #1: I am very confused by Figure 6: ORF1 appears to co-immunoprecipitate equally with all of the immunoprecipitated MOV10 proteins, regardless of whether they were even immunoprecipitated with the anti-Flag antibody. Specifically, the last three MOV10 truncations are not present in the Flag immunoprecipitation, although there is equal ORF1 co-immunoprecipitated. Unless there is a labeling problem, it appears that ORF1 non-specifically associates with the immunoprecipitating antibody. That control should be shown. Specifically, what happens when you immunoprecipitatate LINE-expressing HeLa cells without any Flag-tagged protein?***

***Further, Figure S3 does not support this statement “Our findings demonstrated that deletion of amino acids 966-1003 in MOV10 does not compromise its functional capacity. Specifically, the truncated MOV10 variant maintains the ability to form large cytoplasmic granules and complete activity comparable to the full-length MOV10(S3 Fig).”***

***Figure S3 shows the granule sizes***

**Response:** We appreciate the reviewer for pointing out these mistakes in the manuscript. Indeed, co-immunoprecipitation assays shown in Figure 6 were performed using an anti-ORF1p-specific antibody. We have now corrected the antibody from“Flag” to “anti-ORF1p”in the revised manuscript. In addition, we have corrected “S3 Fig” to “S4 Fig” in line 497.

***Reviewer #2: The authors indicate that anti-Flag antibodies were used for experiments presented in Figure 6, but this does not seem correct. In the methods they indicate that they used either anti-Flag or anti-ORF1p antibodies. Please confirm which antibodies were used for the IP experiments shown in Fig. 6.***

**Response：**We apologize for the mistake. Instead of anti-Flag, anti-ORF1p antibody was used in the co-immunoprecipitation assays in Figure 6. We have corrected “Flag” to “anti-ORF1p” in figure legal and methods.
